# An interactive, multi-modal Anatomy workshop improves academic performance in the health sciences: a cohort study

**DOI:** 10.1186/s12909-016-0541-4

**Published:** 2016-01-12

**Authors:** Leslie L. Nicholson, Darren Reed, Cliffton Chan

**Affiliations:** Discipline of Biomedical Science, Sydney Medical School, The University of Sydney, P.O. Box 170, Lidcombe, NSW 1825 Australia

**Keywords:** Self-confidence, Body painting, Engaged enquiry, Learning styles, Musculoskeletal anatomy, Multi-modal learning, Approaches to learning, Education

## Abstract

**Background:**

Students often strategically adopt surface approaches to learning anatomy in order to pass this necessarily content-heavy subject. The consequence of this approach, without understanding and contextualisation, limits transfer of anatomical knowledge to clinical applications. Encouraging deep approaches to learning is challenging in the current environment of lectures and laboratory-based practica. A novel interactive anatomy workshop was proposed in an attempt to address this issue.

**Methods:**

This workshop comprised of body painting, clay modelling, white-boarding and quizzes, and was undertaken by 66 health science students utilising their preferred learning styles. Performance was measured prior to the workshop at the mid-semester examination and after the workshop at the end-semester examination. Differences between mid- and end-semester performances were calculated and compared between workshop attendees and non-attendees. Baseline, post-workshop and follow-up surveys were administered to identify learning styles, goals for attendance, useful aspects of the workshop and self-confidence ratings.

**Results:**

Workshop attendees significantly improved their performance compared to non-attendees (*p* = 0.001) despite a difference at baseline (*p* = 0.05). Increased self-confidence was reported by the attendees (*p* < 0.001). To optimise their learning, 97 % of attendees reported utilising multi-modal learning styles. Five main goals for participating in the workshop included: understanding, strategic engagement, examination preparation, memorisation and increasing self-confidence. All attendees reported achieving these goals. The most useful components of the workshop were body painting and clay modelling.

**Conclusions:**

This interactive workshop improved attendees’ examination performance and promoted engaged-enquiry and deeper learning. This tool accommodates varied learning styles and improves self-confidence, which may be a valuable supplement to traditional anatomy teaching.

## Background

Anatomy underpins all medical and allied health professional education informing clinical reasoning. Indeed, the consequence of less-than-optimal anatomy education is the graduation of incompetent healthcare professionals [1]. Currently, musculoskeletal anatomy for allied health students (physical, occupational and speech therapists, exercise scientists and physiologists) is predominantly taught in laboratories using prosected cadaveric specimens and in didactic lectures. These invaluable teaching resources have withstood the pedagogical test of time [1].

Today’s anatomy students have less dedicated laboratory time than in years past [2]. The addition of new curricular content, the semesterisation of units of study, the standardisation of the number of units and face-to-face academic to student time, as well as increased student to staff ratios have all led to a decreased contribution of anatomy to the overall education of health professionals [3–5].

Anatomy is traditionally viewed by medical and allied health students as challenging with syllabi necessarily content-heavy [6]. Phenomenographic analysis of medical students’ approaches to anatomical study revealed three means of learning the content - through memorising, contextualising or experiencing [7]. However, the complexity of the subject material and its placement early in the relevant courses results in students strategically adopting surface approaches to learning [8], including memorisation of lists (rote learning) and the use of mnemonics [7]. The student who adopts a predominantly surface approach often fails to find meaning in the new knowledge, engage with it, seek connections or make clinical implications [9]. Conversely, a student who adopts a deep approach to learning seeks to understand the material, find relationships and themes in the content and transfer new concepts to a variety of contexts [9, 10].

To foster deep, meaningful learning in anatomy it is important to ensure vocational relevance [11]. Such an approach encourages students to interpret the new knowledge in the context of real-life, clinically-oriented, albeit novice experiences [9, 10, 12]. Where there is a complex vocabulary associated with learning, a deep approach may require a preliminary stage of rote learning, difficult to distinguish from a surface approach [13]. Indeed, Pandey and Zimitat [5] concluded that anatomy is a discipline area where distinctions between deep and surface learning may be blurred.

Student perceptions of their learning environment influence how they learn [12, 14, 15]. High content loads delivered over single semesters are undoubtedly a factor that negatively affects the student learning experience [9]. The challenge therefore, is to encourage the adoption of a deep approach to learning in a content-dense subject. Research suggests that changing from traditional instructor-dominated pedagogy to a more learner-centred approach may promote deeper levels of understanding and meaning [10, 16, 17]. In addition, Floyd et al. [18] report that elucidating the connection between the course content and the “real world” in the context of the student’s future career, stimulates the students’ perceived value of the course, and consequently strongly encourages engagement with the content and the utilisation of deeper learning strategies [19].

Academic performance may improve when instruction is adapted to student-preferred learning styles [19]. Fleming and Mills [20] defined four sensory modalities of learning: visual (prefer using diagrams, charts, mind-maps), auditory (acquire knowledge through listening), read-write (prefer texts and lists) and kinesthetic (prefer hands-on experience). While some learners predominantly use one of these learning modalities, multimodal learners utilise a combination of these [21]. It is reported that between 54–69 % of first year medical and nursing students from the U.S.A, Australia and the Middle East preferred using multiple modes [22–24]. DiLullo and colleagues [25] further hypothesise that among the various health care professions, students may display unique learning predispositions with similar core learning traits. Ideally, the learning environment of health professionals should not only incorporate a range of learning activities to accommodate learning style preference, but also encourage engaged enquiry of all students. However, there is limited educational research that has tested effective methods of facilitating deeper approaches to anatomical learning and knowledge retention using multi-modal teaching.

The aim of this study was to assess the effect of a novel anatomy learning and teaching tool in the form of an interactive, multi-modal workshop, on academic performance and perceptions of self-confidence using a mixed-methods research design.

## Methods

### Pilot study

A pilot anatomy workshop, with 35 undergraduate anatomy students representative of all participating health science courses, was conducted in Semester 1, 2013. The non-attendees of this cohort numbered 267. Elements of the pilot workshop were based on curricular content while the learning activities incorporated visual and tactile learning techniques not currently utilised in the anatomy units of study [26]. The authors used a range of open and closed questions in a survey to capture students’ feedback on the elements and the practicalities of the workshop and their learning style preferences. The feedback was utilised to refine and optimise the final proposed workshop. Subsequently, the authors discussed revisions and additions to the workshop before a larger cohort of students was invited to participate the following year. Ethical approval to conduct the study was granted by The University of Sydney’s Human Research Ethics Committee (No. 2013/812). All participants provided written consent to participate and the use of their examination results for the study.

### Participants and recruitment

The Semester 1, 2014 cohort of the subject Functional Musculoskeletal Anatomy (upper limb), comprised of 355 students. This mandatory subject is delivered in both lab/tutorial and lecture modes to first-year undergraduate students in the Bachelor level courses of Health Science, Physiotherapy, Occupational Therapy, Exercise and Sport Science and Exercise Physiology. An invitation to participate in an anatomy workshop was communicated at the end of a lecture and via email with 70 positions available. Students registered their interest via email to one of the authors and an automated email to the first 70 students confirmed their place in the workshop.

### Workshop procedures

Based on the feedback from the pilot study, the four-hour workshop was scheduled during the study vacation two weeks prior to their exams. On arrival, the students were oriented to the format and resources available. Students were encouraged to identify areas in which they were less confident and to prioritise the resources on that basis; they did not have to undertake all aspects of the workshop. Three experienced anatomists were available to assist and facilitate student learning.

**Fig. 1:**
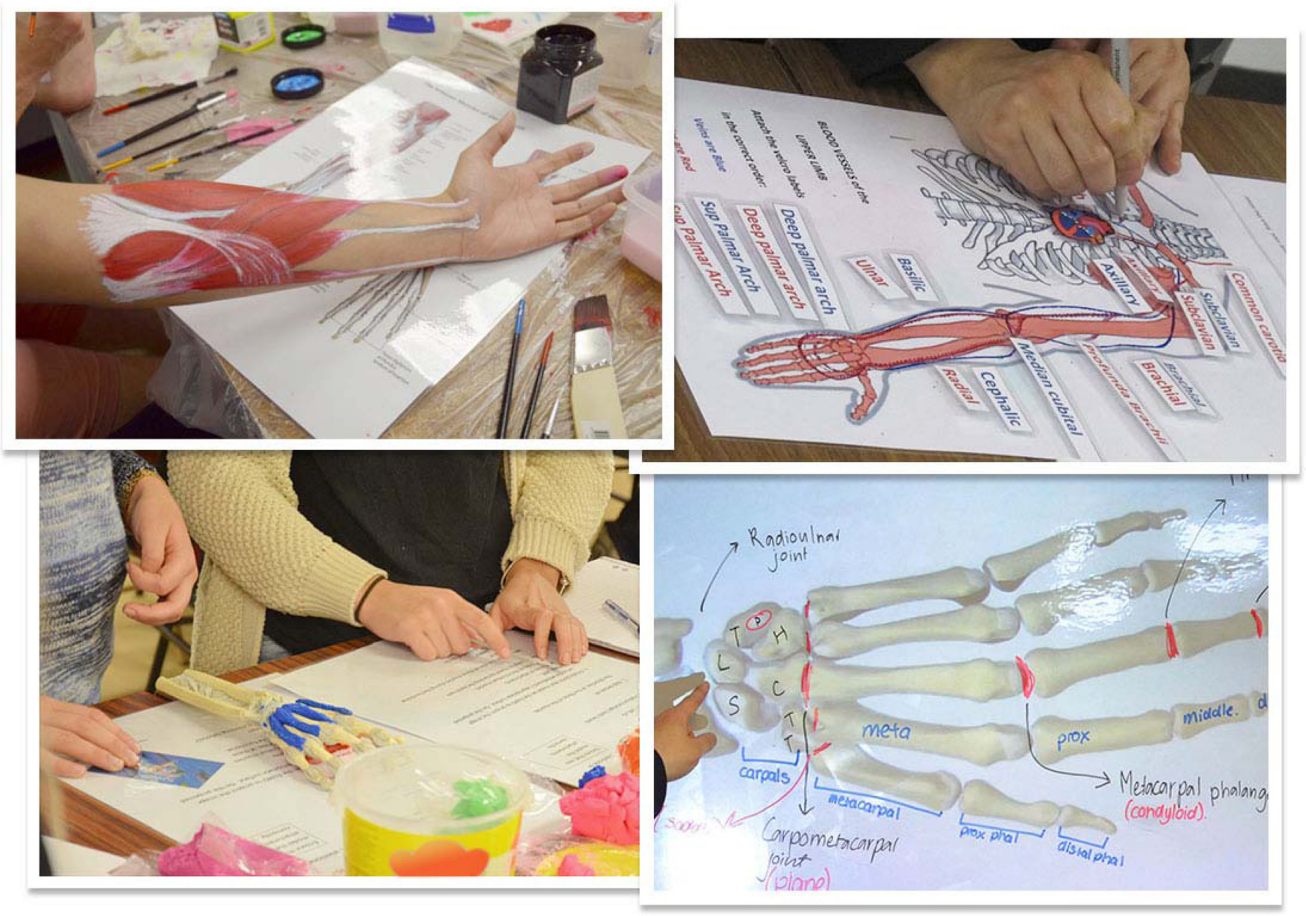
Depiction of the four interactive activities undertaken in the Anatomy Workshop – body painting (top left), quizzing (top right), clay modelling (bottom left) and white-boarding (bottom right)

The workshop was comprised of four interactive activities: body painting, white-boarding, clay modelling and quizzes (Fig. 1). One activity was dedicated to body painting with water-based face and body paints (Derivan Paints). Participants painted deep or superficial bones, muscles and tendons of the upper limbs, spinal and peripheral cutaneous nerve distributions on each other guided by laminated graphics [27]. For the second activity, plastic models and articulated bones of the upper limb were provided for the clay modelling activity. Students were encouraged to use modelling clay to construct muscles of the upper limb, attempting to deduce action and function through precise understanding of muscle attachments in three dimensions. Expertise in body painting and clay modelling was not the focus, but students were encouraged to be detailed in the process rather than being artistically creative in the activities. Four projector systems (Elmo L-1ex Visualisers, Japan) and whiteboards were set up. Here, images in atlases/textbooks, upper limb plastic models, graphics and schematic drawings, were projected onto whiteboards. Once projected, students could label structures, complete diagrams and brainstorm the functional applications of the relevant anatomy. Finally, the fourth activity required participants either individually or in groups to undertake quizzes and to complete tables and schematic figures related to functional and clinical applications. Students were free to move between the four activities during the workshop.

### Outcome measures and statistical analysis

#### Quantitative data

Prior to the workshop, students completed an online initial survey (T0), administered via SurveyMonkey Inc. (Palo Alto, California, USA). The survey was comprised of three elements. Firstly we aimed to gather general demographic information (age, gender, degree). Secondly we assessed students’ perception of their own learning strategies based on the VARK questionnaire 7.1 [28] a valid tool utilised widely in related medical and nursing educational research [23, 24, 29]. Due to the emphasis on read-write learning strategies during earlier and current education and the results of the pilot study, we expect that all students use this strategy and therefore required students to indicate which of the remaining three strategies (visual, kinaesthetic, auditory or a combination of these) they commonly employ. Lastly students rated their self-perceived confidence in seven areas of musculoskeletal anatomy covered in the subject (5-point Likert scale: very not confident [1], not confident [2], neutral [3], confident [4], very confident [5]). In the latter item, the seven areas of upper limb musculoskeletal anatomy included surface anatomy, bone and joint features, muscle attachments and actions, muscle function, myotomes and dermatomes, brachial plexus, nerves and blood vessels.

At the conclusion of the workshop, students immediately completed a paper-based post-workshop survey (T1). This survey evaluated: i) the usefulness of the workshop in consolidating the material learned during the semester (5-point Likert Scale: very useless [1], useless [2], neutral [3], useful [4], very useful [5]), ii) whether their attendance at the workshop achieved their goals stated in the baseline survey (dichotomous response yes/no), iii) their post-workshop self-perceived confidence with the aforementioned seven areas of musculoskeletal anatomy, and iv) a score of overall satisfaction with the Anatomy Workshop (11-point visual analogue scale: waste of time [0], neutral [5], fantastic [10]).

A final follow-up survey (T2) was administered two weeks after the workshop, via the SurveyMonkey website, following their final examinations but prior to receiving any results. This survey assessed the participants’ self-report of the workshop’s usefulness in examination preparations as well as performance, using dichotomous questions (yes/no response).

The entire cohort’s mid-semester examination (MSE) and end-semester examination (ESE) results were collected at the conclusion of the semester. The MSE consisted of a multiple-choice lab-based examination testing identification of anatomical structures and their functional applications. The ESE consisted of two components: An identification examination similar to the MSE covering new content and a multiple-choice theory examination covering the entire semester’s content. All data were entered and analysed in IBM SPSS Statistics for Windows, Version 20.0. Armonk, NY: IBM Corp. Descriptive statistics were undertaken for demographic characteristics and outcome measures. One-factor repeated measure ANOVAs were performed to determine any significant differences in mean MSE, ESE examination performance and change scores (ESE - MSE) between the two groups (attendees and non-attendees). Following this, a post-hoc linear regression analysis was performed to evaluate any differences in the ESE results, whilst taking into account any baseline performance differences between the groups (i.e. mid-semester examination results). The dependent variable was end-semester result (%) and the independent variables were mid-semester result (%) and attendance at the workshop. Finally, the confidence scores of the seven areas of musculoskeletal anatomy were averaged for each attendee at T0 and T1, and then a student’s t-test was undertaken to determine change in confidence between these two time points.

#### Qualitative data

Students were asked about their main goals for attending the workshop in the initial survey (T0) and commented on what components of the Anatomy Workshop they found were the most useful in the post-workshop survey (T1). A comprehensive narrative themes-based analysis was performed on this data, as described by Braun and Clarke [30].

## Results

### Demographic data

**Fig. 2:**
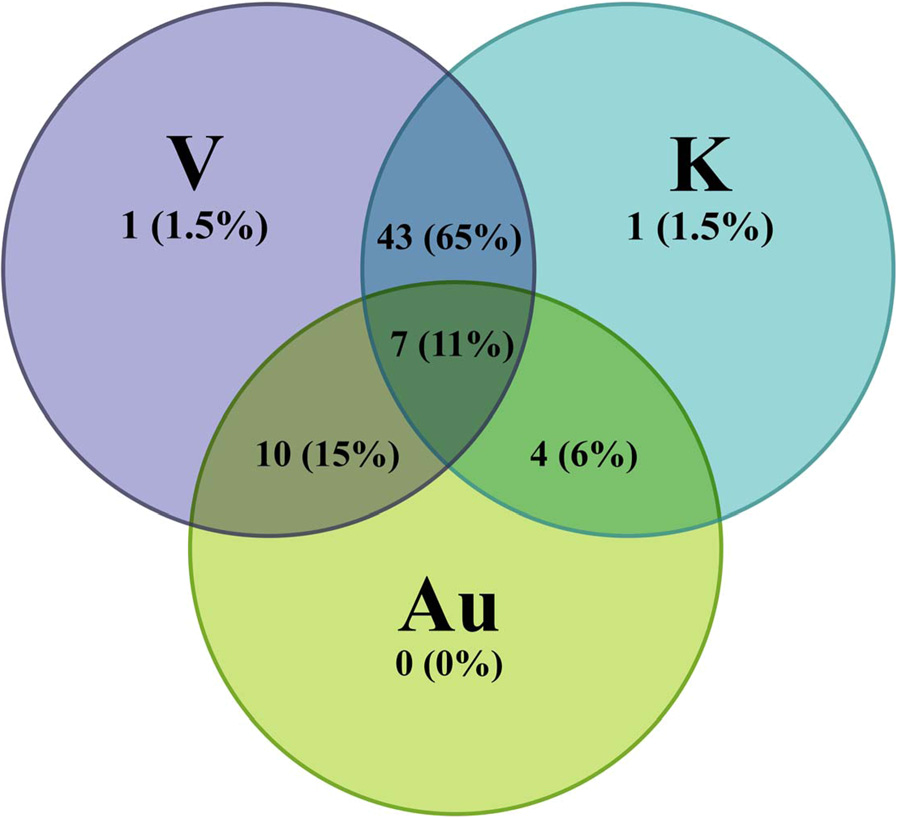
Depiction of the self-identified learning styles, focusing on visual (V), kinaesthetic (K) and auditory (Au) input preferences of the 66 workshop attendees (%). Overlapping sections indicate multi-modal learning preferences

Four students withdrew from the study due to illness leaving 66 students (17 M:49 F, average age 20.0 ± 4.2 years) in attendance. The number of attendees from each of the five bachelor degree courses as a proportion of those enrolled in Functional Musculoskeletal Anatomy A is shown in Table 1. The proportion of participants who self-identified their approaches to study as focusing on visual (V)/ kinaesthetic (K)/ auditory (Au) learning is illustrated in Fig. 2.

**Table 1:** Attendees and non-attendees enrolled in the subject Functional Musculoskeletal Anatomy sorted by degree

Bachelor Degree	Attendees n (% of total)	Non Attendees n (% of total)	Ratio Comparison (Attendees:Non-Attendees)
Exercise Physiology	23 (35 %)	37 (13 %)	1: 0.4
Occupational Therapy	3 (5 %)	10 (4 %)	1: 0.8
Physiotherapy	16 (24 %)	85 (29 %)	1: 1.3
Exercise Sports Science	20 (30 %)	118 (41 %)	1: 1.4
Health Sciences	4 (6 %)	39 (13 %)	1: 2.2
Total	66	289	-

### Pre- and post- workshop examination results

**Fig. 3:**
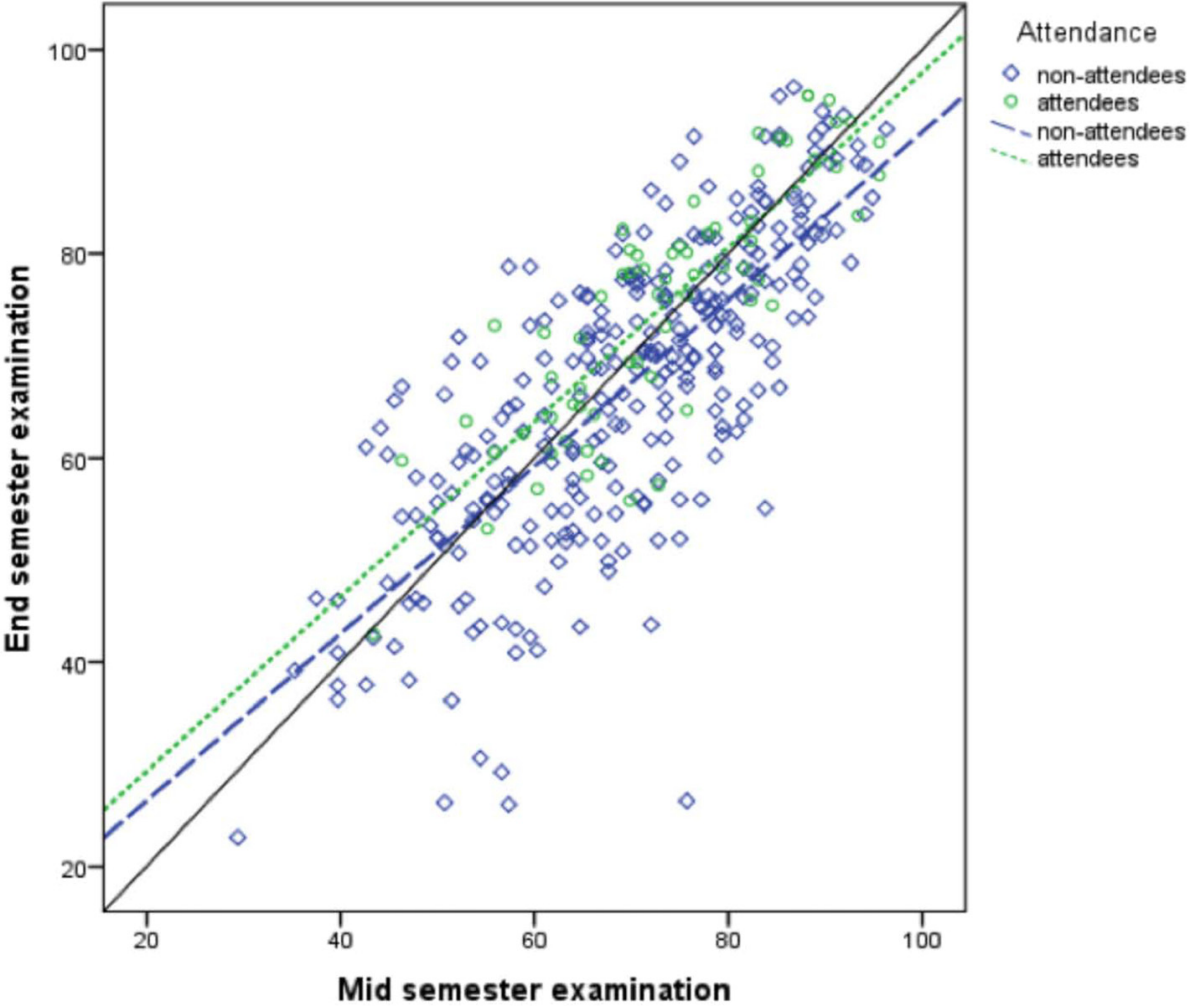
ESE and MSE results of workshop attendees and non-attendees. The solid black line represents perfect coherence (R = 1) between results of the two examinations. The dotted lines represent the line of best fit for the individual results for attendees and non-attendees

The mean (±SD) MSE and ESE results as a percentage for the attendees and non-attendees are reported in Table 2. The results of the one-factor repeated measures ANOVA indicated a significant difference between cohorts at both the MSE (F_1,354_ = 3.9,*p* = 0.05) and ESE (F_1,354_ = 15.83,*p* < 0.001) with greater mean scores in the attendee cohort at both the MSE and ESE. There was also a significant difference in change scores (ESE - MSE) between attendees and non-attendees (F_1,354_ = 10.36, *p* = 0.001) with attendees attaining a positive change score and non-attendees a negative score. Results of the linear regression analysis for the MSE and ESE results are illustrated in Fig. 3, where the line of best fit was R^2^ = 0.711 with slope of the line β = 0.832, 95 % CI [0.699–0.964] for the attendees and R^2^ = 0.569, β = 0.695, 95 % CI [0.625–0.765] for the non-attendees. With the significantly different baseline MSE results taken into consideration by the regression analysis, the different slopes indicate a significantly different change in results (ESE-MSE) between groups, with an increase in mean scores for the attendee cohort and a decrease in the non-attending cohort.

**Table 2:** Mean score of mid-semester examination (MSE) and end-semester examinations (ESE) and percentage change (ESE – MSE) of students

	MSE (% ± SD)	ESE (% ± SD)	% Change ESE-MSE (% ± SD)
Workshop Attendees	73.3 ± 11.8†	74.9 ± 12.0‡	1.6 ± 6.7*
Non-Attendees	69.7 ± 13.6	67.1 ± 14.8	−2.3 ± 10.0

† indicates significantly greater MSE results for attendees (*p* = 0.048), ‡ indicates significantly greater ESE results for attendees (*p* < 0.001), * indicates significant difference in change scores between attendees and non-attendees (*p* = 0.001)

### Ratings of self-confidence and usefulness of the workshops

Participants’ mean self-perceived rating of confidence prior to the workshop (/5 ± SD) was 3.1 ± 0.5 and immediately post workshop was 3.8 ± 0.4. This resulted in a significant increase in self-confidence between the two occasions with mean change 0.7 ± 0.6, 95 % CI [0.58–0.86] (*p* < 0.001). Pearson’s correlation demonstrated no relationship between change in self-confidence following the workshop and the ESE results (*r* = −0.18,*p* = 0.16). Immediately post-workshop, average overall satisfaction with the Anatomy Workshop (/10 ± SD) was rated as 8.5 ± 1.1. Two weeks after the workshop, 59 of 66 (89 % response rate) attendees completed the follow-up (T2) survey. Fifty-six students reported the workshop as useful for their examination preparation and their academic performance, while three reported it was not useful as, ‘it did not teach them anything new’.

### Pre and post workshop feedback

A narrative, themes-based analysis of the qualitative responses revealed five major goals for participants attending the workshop. These themes and examples of individual student comments are summarised in Table 3A. Some students identified multiple goals for attendance. Consequently, reported totals for each goal are calculated as a proportion of the total students attending the workshop and the sum of the five categories does not total 100 %. All participants (100 %) reported that their goals for attending the anatomy workshop, stated in the T0 survey, were attained. Five major themes were identified as the participants’ most useful aspects in attaining their goals. A summary of these themes and examples of participant comments are reported in Table 3B.

**Table 3:** A) Major goals of workshop attendees. B) Most useful aspects of the workshop in attaining attendee’s goals

A) PRE-WORKSHOP SURVEY ‘What are your main goals you would like to achieve from participating in the Anatomy Workshop’	
Themes and Description (%, n/66)	Examples of Student Comments
1. Understanding content (65 %; 43/66)	“T*o achieve a better understanding of the muscles in the body rather than just memorising them….*” (M*ale, 18 years*).
The majority of participants acknowledged that anatomy was more than just memorisation and wanted to engage with the structural anatomy to understand concepts, functional applications and possible clinical relevance.	*“Understanding of functional anatomy and concepts better…” (Male, 18 years)*
	*“Better understanding of concepts in regards to functions of muscles…” (Female, 18 years)*
2. Strategic engagement with content (32 %; 21/66)	“T*o explore creative ways of learning anatomy…” (Female, 27 years*)
Almost a third of the attendees identified the need for and expressed the desire to engage with anatomy through a variety of learning techniques. They sought to supplement their wet-lab anatomy learning with innovative methods not utilised in their regular classes.	*“…to consolidate learning by doing something different.” (Male, 18 years)*
	*“…to get some alternative approaches to the anatomy subject.” (Female, 18 years)*
3. Examination preparation (26 %; 18/66)	*“…revise the content to help me do better in the final exam.” (Female, 42 years)*
Some participants attended the workshop to optimise their examination performance. The workshop was perceived as an opportunity to up-skill in examination technique and undertake structured revision.	*“Learn better exam technique.” (Female, 22 years)*
	*“… improve my grades.” (Female, 18 years)*
4. Memorisation of content (23 %; 15/66)	“…*to better memorise anatomy of the upper limb.” (Female, 38 years*)
Some participants indicated that they attended the workshop in order to aid in short-term retention of the large volume of subject content.	*“…revision of the muscles, ligaments and bones of the body.” (Female, 21 year)*
5. Increase self-confidence (9 %; 6/66)	“…*to be able to confidently identify all muscles and nerves …” (Female, 19 years*) *“To be more confident with every aspect of anatomy.” (Female, 18 years)*
A small portion of participants reported attending the workshop with the goal to increase self-confidence in their anatomical knowledge base. These students perceived that their performance would be enhanced by improving their confidence with the content.	
B) POST-WORKSHOP SURVEY ‘What component of the Anatomy Workshop did you find most useful?’	
Themes and Description (%, n/66)	Example of Student Comments
1. Body painting/ clay modelling (55 %; 36/66)	*“The modelling- great to be able to build muscles…” (Female, 27 years)*
Over half of the attendees stated that the novel experience of body painting was useful to increase their appreciation of anatomy.	*“… painting and clay modelling because visual representation assisted in remembering muscles.” (Female, 19 years)*
2. Tables, schematic drawings and quizzes (46 %; 30/66)	*“The quizzes consolidated my learning through exam style questions.” (Female, 18 years)*
Completing the muscle attachment and function tables, clinically-based cases, drawings of anatomical structures and undertaking timed quizzes were reported as useful for learning for nearly half of the attendees.	*“The questions were very useful in testing my understanding…” (Female, 21 year)*
3. White-boarding (41 %; 27/66)	*“The whiteboards helped to identify attachment sites and bone markings.” (Female, 18 years)*
Attendees reported that the use of white-boarding enhanced and consolidated their learning.	*“…putting everything I know on a board was helpful.” (Female, 18 years)*
4. Group discussion/ peer teaching (14 %; 9/66)	*“…collaborating with other students.” (Female, 18 years)*
Group interaction and collaborative learning, through discussion and teaching their peers during the different activities in the workshop was identified as helpful in clarifying attendees’ anatomical understanding.	*“…interaction with peers.” (Female, 22 years)*
5. Academic staff supervision (9 %; 6/66)	*“Ability to talk to teachers and ask any questions in an open environment.” (Male, 20 year)*
A small portion of attendees indicated that having the opportunity for unconstrained access to experienced academic staff was beneficial.	*“Was really helpful when a tutor was at the station to talk you through exactly what was happening…” (Female, 19 years)*

## Discussion

Undertaking this innovative, interactive workshop significantly improved the examination performance of attendees compared to non-attendees. Performance in the subject Functional Musculoskeletal Anatomy of the Upper Limb generally declines between the mid- and end-semester examinations, possibly due to the increased content and complexity of the material covered in the latter half of the semester. The average decrease in examination scores from mid-semester to end-semester over the preceding six semesters was 3.9 %. Consistent with this trend, the average decrement of those students who did not attend the workshop was 2.3 % across the semester. Conversely, the average increment of attendees was 1.6 %. In a competitive educational environment, where failing a core subject incurs emotional, financial and course progression penalties, the seemingly small improvements in examination performance resulting from participation in the workshop are valuable. Therefore, this study supports consideration of the implementation of similar types of workshops as part of core curriculum in undergraduate anatomy units of study.

The aim of the workshop was to determine whether teaching anatomy curricular content using student-centred approaches, such as optimising their engagement and learning experiences and accommodating their preferred learning styles, improved examination performance and self-confidence. Ninety-seven percent of workshop participants perceived that their learning was supported by a combination of at least two styles, with 84 % reporting a preference for kinaesthetically conferred information. This finding concurs with that of other relevant learning style studies. The majority (58 %) of Jordanian third-year nursing students [22], 64 % of American medical students [24] and 80 % of Australian first-year nursing and midwifery students [31] reported multi-modal learning preferences. Such a finding justifies supplementing didactic and wet-lab based teaching with visual anatomical representations (both two and/or three dimensional) and hands-on kinaesthetic experience with body-painting and modelling whilst elucidating the connection between anatomy and the “real world” context of the student’s future career. Importantly, the positive findings of this study may be due to the integration of all learning styles in a conducive learning environment of peer teaching and group participation with academic facilitation.

As anatomy underpins all healthcare, the purpose of anatomy as core learning for healthcare professionals is to provide the student with tools to assist clinical reasoning. Spatial ability or appreciating the spatial orientation of the various anatomical structures is an essential skill for health professionals. This spatial ability, practised by the body painting and modelling components in the workshop, compliments and value-adds to existing teaching on three-dimensional cadavers, potted specimens, living and plastic models as well as two-dimensional imaging and texts/atlases, which can be transferred to the diagnosis and management of the patient. The clinically case-oriented quizzes and table/figure completion tasks facilitated the blending of academic learning with real-world applications, assisting the student to situate new knowledge with cognitive schemas to be recalled in later academic and clinical scenarios [25].

Students’ self-perceived confidence significantly improved as a result of undertaking the workshop. It was hypothesised that greater self-confidence would result in an improvement in academic performance [32]. The findings of the current study do not support this expectation, as the increased confidence did not translate to significant mean improvements from mid- to end-semester examination performance. A possible reason for the lack of association found here may be that the measure of confidence was not specific enough to the performance assessment. Feeling confident with their anatomy knowledge may not assist the student in exam technique. Pajares [33] explains that self-efficacy, highly correlated to self-confidence, is only one determinant of academic performance, citing anxiety, self-regulation and mental ability as other significant determinants. Indeed, in their meta-analysis of 39 studies of the relationship of self-efficacy to academic performance, Multon and colleagues [34] reported that self-efficacy accounted for only 14 % of the variance in academic performance. Future research into interventions to improve academic performance should incorporate these multiple determinants and identify which have the most profound effect.

Narrative themes-based analysis revealed that the majority of participants (66 %) desired to understand and contextualise anatomy. Only a minority (23 %) viewed memorisation of content as a desired goal. With all attendees reporting achievement of their goals for attending, the workshop was successful in facilitating students to better understand anatomy, engage with the content, memorise important anatomy facts as well as aid students to prepare for their examination. Attendees appreciated the importance of not only memorising the content for examination purposes, but rather the relevance and need for anatomy to function as clinical practitioners [6].

Body painting and clay modelling were reported to be the most useful components of the anatomy workshop, closely followed by the quizzes and white-boarding. Previous research has suggested the usefulness of body painting and clay modelling in increasing student engagement as well as consolidation of anatomy [35–37]. Not only would the findings from this study support these claims, but that it must be highlighted that these learning tools would be valuable for healthcare students whose preferred learning style involves visualisation and kinaesthesis. Some students reported that the quizzes helped them to identify areas of knowledge deficit and to consolidate existing knowledge. The academics who facilitated the workshop reported that group participation, particularly in the quizzes and white-boarding, was considerably more interactive than in wet-lab sessions. Students engaged with the content and with each other, taking on the roles of educators and peer learners. By assuming the responsibility of teaching their peers, students not only consolidate course content, but also develop communication skills, teamwork, leadership and respect for peers that are vital to developing professionalism early in healthcare careers [38].

### Limitations

One of the limitations to this study was the self-selection recruitment design. It may be argued that more goal-oriented and academically committed students enrolled for the workshop. However, the effect of such selection bias would not affect the overall findings of this study. Although there was a difference in exam performance between attendees and non-attendees at inception, the regression analysis factored this difference into consideration, finding that the difference in performance between examinations was significant. Further, all students had identical access to curricular content including live and recorded lectures, practical lab classes, on-line quizzes, museum-potted specimens and resources, surface anatomy and radiology iBooks, frequently-asked-question files, Facebook and Blackboard discussion forums and open access to tutors and lecturers, similar to that reported by Diaz and Woolley [26]. Those who did not participate in the workshop were free to supplement their anatomy learning using any or all of these depending on their learning preferences. If they perceived that they were disadvantaged by missing out on the workshop there were many avenues to support their further study. The difference in the educational experience for the Semester 1, 2014 cohort was therefore participation in the 4 h workshop.

It is possible that the potential for post-workshop improvement was less for high performing students, resulting in a ceiling effect of the workshop on ESE results. Had students who performed worse on the MSE enrolled, there is a possibility that the effect of the workshop on examination performance would have been greater still. Another limitation is the questionable validity of the VARK as a self-assessment tool for learning traits due to item wording and the scale’s scoring algorithms [39]. However there is preliminary evidence to support its use in educational research and further investigation is warranted.

### Future recommendations

Future studies should incept a larger experimental cohort, case-matched for course, age, gender and importantly for baseline examination performance. The investigation of self-efficacy as opposed to self-confidence could be useful to better determine the relationship between self-efficacy and performance. Further research is needed to determine whether such a workshop would be beneficial to the academic performance of all students. In addition to access for all enrolled students, to enhance the cost-effectiveness and perceived value of a workshop, a nominal fee to students may be an option if the workshop remains extra-curricular [40]. Finally, scheduling a second workshop in the later years of medical and healthcare study may enhance longer-term knowledge retention. This may be useful revision for clinical practica, but will require further investigation.

## Conclusions

The workshop as conducted in this study improves examination performance and self-confidence and promotes engaged enquiry and deep learning with integration of anatomy into the real-life clinical context. Assisting in development of generic professional attributes, the workshop may be a valuable addition to traditional anatomy learning and teaching in the health sciences.
